# Can natural killer cell activity help screen patients requiring a biopsy for the diagnosis of prostate cancer?

**DOI:** 10.1590/S1677-5538.IBJU.2019.0268

**Published:** 2020-01-10

**Authors:** Bum Sik Tae, Byeong Jo Jeon, Young Hoon Lee, Hoon Choi, Jae Young Park, Jae Hyun Bae

**Affiliations:** 1 Korea University Ansan Hospital Korea University College of Medicine Ansan Korea Department of Urology, Korea University Ansan Hospital, Korea University College of Medicine, Ansan, Korea

**Keywords:** Prostate neoplasm, Killer Cells, Natural, Mass Screening

## Abstract

**Purpose:**

To evaluate the usefulness of natural killer cell activity (NKA) in diagnosing prostate cancer (PC).

**Materials and Methods:**

The medical records of patients who underwent transrectal prostate biopsy (TRBx) at Korea University Ansan Hospital between May 2017 and December 2017 were retrospectively reviewed. NKA levels were measured using NK Vue® Tubes (ATgen, Sungnam, Korea). All blood samples were obtained at 8 AM on the day of biopsy. Patients with other malignancies, chronic inflammatory conditions, high prostate-specific antigen (PSA) level (>20ng/mL), or history of taking 5-alpha-reductase inhibitor or testosterone replacement therapy were excluded.

**Results:**

A total of 102 patients who underwent TRBx for PC diagnosis were enrolled. Among them, 50 were diagnosed with PC. Significant differences in age and NKA level were observed between the PC and no-PC groups. Receiver operating characteristic (ROC) curve analysis showed that the optimal cut-off of NKA level for the prediction of PC was 500pg/dL, with a sensitivity of 68.0% and a specificity of 73.1%. In addition, NKA level (0.630) had the greatest area under the ROC curve compared to those for the ratio of total PSA to free PSA (0.597) and PSA density (0.578).

**Conclusions:**

The results of this pilot study revealed that low NKA and high PSA levels were likely to be associated with a positive TRBx outcome. NKA detection was easy and improved the diagnostic accuracy of PC.

## INTRODUCTION

Prostate cancer (PC) is one of the most common malignancies in Korean men (1). The prostate-specific antigen (PSA) level is widely used in the diagnosis and follow-up of PC, and the increase in the incidence of PC has been attributed to the widespread testing of PSA level in daily clinical practice (2). Depending on each clinician, patients with PSA levels >3.5-4.0ng/mL may be recommended to undergo prostate biopsy. However, although PSA is an useful biomarker for the diagnosis of PC, its specificity is low and significantly high-risk PC cases cannot be detected based on PSA levels (3). In addition, the American Cancer Society reported that the sensitivity of PSA for PC detection is only 21%, whereas the specificity is 91% when the normal PSA level is defined as <4.0ng/mL (4). Although transrectal prostate biopsy (TRBx) is the most widely used method to diagnose PC, it may lead to various complications; therefore, patients should be carefully selected (5, 6).

To overcome the current limitations, various biomarkers have been evaluated to improve the diagnostic accuracy of PC (7-9). However, most of these novel markers had limitations of difficulty in assessment, expense, and additional intervention. However, increasing attention has been focused on natural killer (NK) cells in cancer settings as they are involved in innate immunity and play a major role in anticancer mechanisms (10, 11). Previous studies reported a correlation between low NK cell level and PC stage, prognosis, and treatment response (12, 13). However, some conflicting issues exist regarding the use of NK cell activity (NKA) as a marker for PC detection and screening. In their pilot study, Barkin et al. first reported that patients with low NKA levels (<200pg/mL) were more likely to have a positive outcome at prostate biopsy (14). However, Song et al. reported that NKA was not very useful for the detection of PC and prediction of Gleason grade (15). To address this issue, we measured the NKA levels of patients with suspected PC who underwent transrectal ultrasonography-guided prostate biopsy and evaluated the clinical usefulness of NKA in diagnosing this cancer.

## MATERIALS AND METHODS

The institutional review board of Korea University Ansan Hospital approved this study’s protocol (IRB No. 2019AS0060). This study was conducted in accordance with the guidelines of the Declaration of Helsinki. Data collected from patients who visited the Department of Urology between May 2017 and May 2018 were retrospectively evaluated. Men aged ≥18 years with elevated PSA levels (>2.5ng/mL) and/or abnormal findings on digital rectal examination were enrolled in our analysis. The exclusion criteria were as follows: (1) chronic inflammatory condition requiring anti-inflammatory treatment, (2) other medical conditions that might affect immune response (e.g., ulcerative colitis, Crohn’s disease, lupus, or other connective tissue diseases), (3) medical history of 5-alpha reductase inhibitor use, (4) history of testosterone replacement treatment, and (5) history of other cancers.

### NKA measurement

NKA levels were measured as described by Song et al. (15). One milliliter of whole blood was collected using NK Vue® Tubes (ATgen, Sungnam, Korea). All blood samples were obtained at 8 AM on the day of biopsy. The collected samples were incubated at 37°C for 24h under 5% CO_2_ with the indicated dose of Promoca™ (ATGen, Seongnam-si, Korea) and 1mL of RPMI 1640 medium (Cellgro/Mediatech, Manassas, VA, USA). The supernatant was then collected, centrifuged at 11.500 x g for 5min., collected in another conical 1.5mL Eppendorf tube, and immediately loaded onto the enzyme-linked immunosorbent assay (ELISA) plates. During the 24h incubation at 37°C, cytokine (Promoca) stimulated the NKA and levels of interferon (IFN)-g secreted into the plasma was quantitated by ELISA. IFN-g was measured in pg/mL and the detection/sensitivity range of the ELISA was 40-2.000pg/mL.

### Statistical Analysis

The patients were divided into two groups according to the presence or absence of PC, and NKA levels between the two groups were compared by non-parametric Wilcoxon-Mann-Whitney tests. The diagnostic test performance of NKA was evaluated by receiver operating characteristic (ROC) curve analysis. Youden’s index was calculated to quote the concentration at which the sum of sensitivity and specificity is maximized. In addition, ROC curves were compared for the diagnostic performance of NKA, ratio of total PSA to free PSA (FTR), and PSA density (PSAD). The sensitivity, specificity, and positive and negative values of the PC marker confirmed by prostate biopsy were calculated at a cut-off value of 500pg/dL. The cut-off points for the calculated biomarker coefficients (NKA, FTR, and PSAD) were determined using MedCalc® (MedCalc Software, Mariakerke, Belgium). Data were analyzed using IBM SPSS Statistics for Windows, version 21.0 (Armonk, NY, USA). P-value <0.05 were considered statistically significant.

## RESULTS

A total of 102 patients who underwent prostate biopsy for suspected PC were included. Among them, 50 were diagnosed with PC. The baseline characteristics of all patients are listed in Table-1. Significant differences in age (mean 61.30±10.49 vs. 65.52±7.35 years, p=0.03) and NKA level (p <0.01) were observed between the PC and no-PC groups. However, there were no significant differences in PSA levels (mean 7.19±3.86 vs. 7.96±4.24ng/mL, p=0.54), prostate volume (mean, 45.97±28.58 vs. 39.82±14.58mL, p=0.06), or free PSA levels (mean 0.98±0.73 vs. 1.27±1.02, p=0.397).

**Table 1:** Baseline characteristics of the studied population (n = 102).

	Total	No tumor group	Tumor Group	P value
**No. of patients (%)**	102	52	50	
**Age (year, SD)**	63.37±9.29	61.30±10.49	65.52±7.35	0.032
**Prostate volume (mL)**	42.96±22.92	45.97±28.58	39.82±14.58	0.058
**Positive DRE**	27 (26.5%)	4 (7.7%)	23 (46.0%)	0.001
**Serum PSA (ng/mL)**				
Mean, SD	7.57±4.05	7.19±3.86	7.96±4.24	0.539
<4 ng/mL [no. (%)]	16 (15.7%)	6 (11.5%)	10 (20.0%)	0.463
≥4 ng/mL [no. (%)]	62 (60.8%)	34 (65.4%)	28 (56.0%)	
≥10 ng/mL [no. (%)]	24 (23.5%)	12 (23.1%)	12 (24.0%)	
**PSA density (ng/mL/mL)**				**0.357**
<0.15 [no. (%)]	42 (41.2%)	20 (38.5%)	22 (44.0%)	
≥0.15 [no. (%)]	60 (58.8%)	32 (61.5%)	28 (56.0%)	
Free PSA	1.12±0.89	0.98±0.73	1.27±1.02	0.397
**NKA**				**0.001**
<200	30 (29.4%)	12 (23.1%)	18 (36.0%)	
200-500	18 (17.6%)	2 (3.8%)	16 (32.0%)	
>500	54 (52.9%)	38 (73.1%)	16 (32.0%)	
**Gleason sum at biopsy**				
6 [no. (%)]	24 (23.5%)	-	24 (48.0%)	
7 [no. (%)]	8 (7.8%)	-	8 (16.0%)	
≥8 [no. (%)]	18 (17.6%)	-	18 (36.0%)	

**DRE =** digital rectal exam; **PSA =** prostate specific antigen, **SD:** standard deviation

The baseline characteristics according to NKA level are described in Table-2. Significant differences in free PSA and prostate volumes were observed according to NKA level. The NKA <200 and NKA <500 groups had higher positive rates for PC (60% vs. 88.9% vs. 20.0% vs. 35.3%, respectively; p <0.001). However, no significant differences in age, proportion of positive digital rectal exam (DRE), and serum PSA level were observed among the four groups.

**Table 2:** Baseline characteristics according to NKA (n = 102).

	NKA<200	200≤NKA<500	500≤NKA<1000	1000≤NKA	P value
**No. of patients (%)**	30	18	20	34	
**Age (year, SD)**	62.93±7.62	66.11±8.49	60.50±10.42	64.06±10.17	0.478
**Prostate volume (mL)**	42.35±15.43	35.80±9.92	45.05±17.62	46.06±33.58	0.041
**Positive DRE**	8 (26.7%)	7 (38.9%)	2 (10.0%)	10 (29.4%)	0.183
**Serum PSA (ng/mL) Mean, SD**	7.96±4.19	8.10±4.62	6.99±2.86	7.28±4.29	0.335
**Serum PSA (ng/mL)**					**0.429**
<4 ng/mL [no. (%)]	4 (13.3%)	4 (22.2%)	2 (10.0%)	6 (17.6%)	
≥4 ng/mL [no. (%)]	22 (73.3%)	8 (44.4%)	14 (70.0%)	18 (52.9%)	
≥10 ng/mL [no. (%)]	4 (13.3%)	6 (33.3%)	4 (20.0%)	10 (29.4%)	
**PSA density (ng/mL/mL)**					**0.437**
<0.15 [no. (%)]	16 (53.3%)	8 (44.4%)	14 (70.0%)	18 (52.9%)	
≥0.15 [no. (%)]	14 (46.7%)	10 (55.6%)	6 (30.0%)	16 (47.1%)	
Free PSA	1.23±0.88	1.24±1.38	0.92±0.43	1.09±0.78	0.036
**Prostate cancer [no. (%)]**	18 (60.0%)	16 (88.9%)	4 (20.0%)	12 (35.3%)	<0.001
**Gleason sum at biopsy**					**<0.001**
No tumor	14 (46.7%)	4 (22.2%)	16 (80.0%)	22 (64.7%)	
6 [no. (%)]	6 (20.0%)	4 (22.2%)	4 (20.0%)	6 (17.6%)	
7 [no. (%)]	4 (13.3%)	0 (0%)	0 (0%)	4 (11.8%)	
≥8 [no. (%)]	6 (20.0%)	10 (55.6%)	0 (0%)	2 (5.9%)	

The absolute risk of diagnosing PC at different NKA levels was calculated and shown in Table-3. Among the 48 patients biopsied with NKA levels below the 500pg/mL cut-off, 70.8% (34/48) were diagnosed with PC with 68.0% sensitivity, 73.1% specificity, and 70.4% negative predictive value. However, among the 30 patients with NKA levels below the 200pg/mL cut-off, 60.0% (18/30) were diagnosed with PC, with a sensitivity of 12.0%, specificity of 76.9%, and negative predictive value of 55.6%. The risks of PC in patients with PSA levels between 2.5 and 10.0ng/mL or between 10.0 and 20.0ng/mL at different cut-off NKA values are also summarized in Table-3.

**Table 3:** Sensitivity and specificity for the NK cell-cutoff.

		Sensitivity (95% CI)	Specificity (95% CI)	Positive predictive value	Negative predictive value
All patients	NKA <200	12.0% (4.5-24.3)	76.9% (63.2-87.5)	60.0%	55.6%
	NKA <500	68.0% (53.3-80.5)	73.1% (59.0-84.4)	70.8%	70.4%
	NKA <1000	76.0% (61.8-86.9)	46.2% (32.2-60.5)	55.9%	64.7%

PSA<10	NKA <200	15.8% (6.0-31.3)	75.0% (58.8-87.3)	61.5%	57.7%
	NKA <500	68.4% (51.3-82.5)	70.0% (53.5-83.4)	68.4%	70.0%
	NKA <1000	79.0% (62.7-90.4)	40.0% (24.9-56.7)	55.6%	66.7%

PSA≥10	NKA <200	16.7% (2.1-48.4)	83.3% (51.6-97.9)	50.0%	50.0%
	NKA <500	66.7% (34.9-90.1)	83.3% (51.6-97.9)	80.0%	71.4%
	NKA <1000	66.7% (34.9-97.9)	33.3% (9.9-65.1)	57.1%	60.0%

**NKA =** Natural killer cell activity

Univariate analysis revealed that FTR (odds ratio [OR] 2.437, p=0.031), positive DRE (OR 10.222, p=0.001), and NKA (<500, OR 5.768, p=0.001) were significant predictive determinants of PC Table-4. However, PSAD was not an independent prognostic factor of PC in logistic regression analysis. Positive DRE (OR 28.437, p=0.001), NKA (<500, OR 8.400, p=0.001), and FTR (OR 3.269, p=0.040) remained significant predictors of PC in the multivariate analysis.

**Table 4:** Univariate and multivariate analyses of parameter for predicting prostate cancer.

	Univariate analysis		Multivariate analysis	

	OR (95% CI)	P value	OR (95% CI)	P value
PSAD >0.15	0.795 (0.361-1.753)	0.570	1.215 (0.419-3.525)	0.582
FTR <0.10	2.437 (1.085-5.474)	0.031	3.269 (1.058-10.264)	0.040
DRE	10.222 (3.199-32.666)	0.001	12.626 (3.452-46.177)	0.001
NKA <500	5.768 (2.457-13.543)	0.010	7.547 (2.717-20.964)	0.001

**PSAD =** PSA density (value 0.15<); **FTR =** ratio of total PSA to free PSA (<0.10); **NKA =** Natural killer cell activity

Univariate analysis revealed that FTR (OR 3.600.031), PSAD (OR 2.941, p=0.049), and NKA (<500, OR 13.000, p=0.001) were significant predictive determinants of a high risk of PC Table-5. However, positive DRE was not an independent prognostic factor of PC in the logistic regression analysis. PSAD (OR 8.433, p=0.013), NKA (<500, OR 10.275, p=0.004), and FTR (OR 11.659, p=0.004) remained significant predictors of a high risk of PC in multivariate analysis.

**Table 5:** Univariate and multivariate analyses for the association of covariates with high risk of prostate cancer.

	Univariate analysis		Multivariate analysis	

	OR (95% CI)	P value	OR (95% CI)	P value
PSAD >0.15	2.941 (1.006-8.596)	0.049	8.433 (1.574-45.187)	0.013
FTR <0.10	3.600 (1.226-10.567)	0.020	11.659 (2.214-61.393)	0.004
DRE	0.667 (0.222-1.998)	0.469	(-)	(-)
NKA <500	13.000 (2.802-60.308)	0.001	10.275 (2.076-50.852)	0.004

**PSAD =** PSA density (value 0.15<); **FTR =** ratio of total PSA to free PSA (<0.10); **NKA =** Natural killer cell activity.

**Figure 1:**
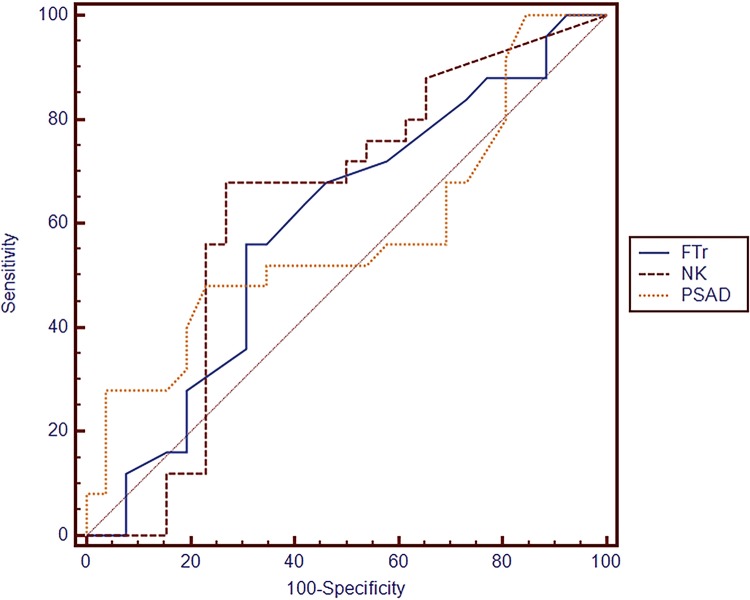
Diagnostic criteria of the receiver operating characteristic (ROC) curve for the tested parameters. **NK =** Natural killer cell activity; **FTr =** ratio of total PSA to free PSA; **PSAD =** PSA density.

The estimated area under the ROC curve of NKA was 63.0% (95 confidence interval [CI]: 52.9-72.4%) Figure-1. However, this area was not significantly higher than that obtained for PSAD (p=0.520) (57.8%, 95% CI: 47.7-67.6%). Moreover, NKA was not significantly superior to FTR (p=0.679), with an area under the ROC curve of 59.7% (95% CI: 49.5-69.3%) for PC diagnosis. Finally, the Youden’s index for NKA in this cohort was 500pg/dL.

## DISCUSSION

PSA is the most commonly used serologic marker for PC screening. Patients with PSA levels ≥3.5 are recommended to undergo prostate biopsy, although criteria differ depending on the clinician. However, PSA is not a perfect marker; therefore, many patients undergo unnecessary prostate biopsy (16). To overcome this limitation, researchers have developed various models using PSA, including FTR:PSA velocity and doubling time or PSAD (17, 18).

NK cells play an important role in tumor immunosurveillance because they activate both the innate and adaptive immune responses in reaction to tumor cells, even without surface antigens (19). Furthermore, various activating and inhibitory receptors on the cell surface regulate NKA, thereby initiating cytolytic processes in tumor cells and avoiding tissue damage (10). Thus, the role of NK cells in suppressing the progression of various tumors is becoming increasingly apparent (20, 21). Their roles in patients with metastatic PC have also been extensively evaluated (22, 23). However, only two studies to date have investigated the efficacy of NKA as a screening tool for PC, with conflicting results (14, 15).

Among the 102 patients enrolled in the present study, 50 (49.0%) were diagnosed with PC. When the patients were divided into two groups according to the presence of PC, no significant difference was observed in the NKA value (P=0.001). Among the 50 patients with PC, the NKA value did not differ significantly according to the Gleason grade (data not shown). These results are inconsistent with those of Song et al. (15). In addition, this study showed that NKA had the highest odds ratio for PC using a cut-off of 500pg/mL. These findings are consistent with those of Barkin et al., however, unlike the previous study that reported NKA values of <200pg/mL, this study presented a higher cut-off value. The reason for the different results among previous studies may be that NKA values differ depending on the measurement time and environment (24). The previous two studies did not mention the blood sampling time of NKA measurements. To reduce bias in the present study, blood sampling was performed between 7 and 8 AM on the day of prostate biopsy.

Several studies have been conducted to assess methods for avoiding unnecessary prostate biopsy. The European Association of Urology (EAU) guidelines indicate the potential prognostic role of serum testing or parameters using PSA in detecting PC (6). Among them, PSAD and FTR are representative and may be clinically helpful. In the present study, NKA differed significantly between the established parameters and showed higher diagnostic accuracy for PC in the ROC curve comparison.

Our results suggest that NKA value can be useful for PC screening in patients who underwent prostate biopsy. However, several limitations must be overcome for NKA to have a diagnostic role in PC screening. First, NK cells are phenotypically and functionally heterogeneous; thus, the reference intervals of NKA values may vary in different populations (25). The patients selected in this study were from a single institution, therefore, further multinational or multicenter investigations with large sample sizes are required. Second, the biological activity of the NK cells is dependent on the integration of signal transducer and activator of transcription signals following stimulation by cytokines such as interleukin (IL)-12 and IL-18, whereas the signal transducer and activator of transcription (STAT) expression within the NK cells may differ among individuals based on various parameters (26). Therefore, the responsiveness of NKA may vary under cytokine stimulation due to differences in various intracellular STAT concentrations (25).

Song et al. showed that prostatic tissues are not properly infiltrated by NK cells and observed a low frequency of NK cells in both normal prostate and PC tissues (27). In addition, CD56^dim^ cells are often dominant in the peripheral blood, whereas CD56^bright^ cells are significantly abundant in tissues, regardless of PC invasion (15). Therefore, Song et al. assumed that the NKA values in patients with localized PC did not differ significantly from those in healthy persons.

Despite these limitations, the strengths of the present study include corroboration of the finding by Barkin et al. that previously-assessed NKA is valuable in PC screening, although our study was conducted in a different race. Second, in contrast to those of Song et al., our findings suggested that NKA may be helpful in screening Korean patients requiring prostate biopsy. Finally, to minimize variations in daytime NKA levels, sampling was performed at the same time for all patients.

## CONCLUSIONS

The results of this pilot study revealed the association between low NKA and high PSA levels and positive outcome of TRBx. NKA was easily detected and improved the diagnostic accuracy of PC. Large multi-institutional prospective studies are required to validate the role of NKA in PC diagnosis.
